# Global trends and frontiers in research on coronary microvascular dysfunction: a bibliometric analysis from 2002 to 2022

**DOI:** 10.1186/s40001-022-00869-8

**Published:** 2022-11-05

**Authors:** Jing Gao, Tiantian Meng, Min Li, Ruolin Du, Jingyi Ding, Anqi Li, Shanshan Yu, Yixiang Li, Qingyong He

**Affiliations:** 1 Department of Cardiology, Guang’anmen Hospital, China Academy of Chinese Medical Sciences, Beijing, China; 2 Graduate School, Beijing University of Chinese Medicine, Beijing, China; 3 Graduate School, Henan University of Chinese Medicine, Zhengzhou, Henan China

**Keywords:** Coronary microvascular dysfunction, Bibliometric analysis, VOSviewer, CiteSpace, Visual analysis

## Abstract

**Background:**

Coronary microvascular dysfunction (CMD) is a leading cause of ischemic heart disease. Over the past few decades, considerable progress has been made with respect to research on CMD. The present study summarized the current research hotspots and trends on CMD by applying a bibliometric approach.

**Methods:**

Relevant publications between 2002 and 2022 were extracted from the Web of Science Core Collection. Visualization network maps of countries, institutions, authors, and co-cited authors were built using VOSviewer. CiteSpace was used for keyword analysis and the construction of a dual-map overlay of journals and a timeline view of co-cited references.

**Results:**

1539 CMD-related publications were extracted for bibliometric analysis. The annual publications generally showed an upward trend. The United States of America was the most prolific country, with 515 publications (33.5%). Camici P. G. was the most influential author, whereas the *European Heart Journal*, *Circulation*, and *Journal of the American College of Cardiology* were the most authoritative journals. Research hotspot analysis revealed that endothelial dysfunction as well as reduced nitric oxide production or bioavailability played critical roles in CMD development. Positron emission tomography was the most widely used imaging method for diagnosis. In addition, microvascular angina, hypertrophic cardiomyopathy, and heart failure have attracted much attention as the main clinical implications. Furthermore, international standards for CMD diagnosis and management may be the future research directions.

**Conclusions:**

This study offers a comprehensive view about the hotspots and development trends of CMD, which can assist subsequent researchers and guide future directions.

**Supplementary Information:**

The online version contains supplementary material available at 10.1186/s40001-022-00869-8.

## Introduction

Coronary microvascular dysfunction (CMD) is a major cause of ischemic heart disease, which is a leading cause of mortality and disability worldwide due to population aging [1, 2]. The coronary microcirculation is a vascular lumen comprising vessels with a diameter < 500 μm, including pre-arterioles, arteries, and capillaries, which plays a crucial role in the physiological regulation of cardiac perfusion [3]. In the presence of enhanced myocardial oxygen demand, the coronary microcirculation can rapidly increase the blood flow via modulation of vascular resistance [4]. Nonetheless, various factors such as inflammation and endothelial dysfunction can lead to functional and structural abnormalities in coronary microvessels. These result in pathological impaired dilatation or increased constriction, ultimately impairing the ability of myocardial blood flow to adjust to variations in myocardial oxygen demand and thereby leading to impaired myocardial perfusion and ischemia; this anomaly is commonly referred to as CMD [5–7]. In the clinical setting, CMD is generally closely related to the pathogenesis and progression of numerous cardiovascular diseases, including microvascular angina, takotsubo syndrome, hypertrophic cardiomyopathy, and heart failure, particularly heart failure with preserved ejection fraction (HFpEF) [8–10]. The prevalence of CMD has been reported by some studies to range from 40 to 70% in microvascular angina and to be 75% in HFpEF [11–13]. In addition, CMD is strongly associated with an increased risk of major adverse cardiovascular events (MACEs), including mortality, heart failure, arrhythmia, and nonfatal myocardial infarction [14].

Over the past few decades, considerable progress has been made with respect to research on CMD, especially in terms of epidemiology, pathophysiology, clinical manifestations, diagnostic methods, prognosis, and management. However, because CMD involves multiple disciplines and fields, gaining systematic and comprehensive knowledge about the current state of research is difficult. Bibliometrics is a discipline that combines mathematics and statistics to evaluate research hotspots and trends in a field through quantitative and qualitative analyses and has been widely used in numerous areas, such as oncology, endocrinology, and rheumatology [15–18]. Nevertheless, no prior bibliometric analysis of CMD has been published. Therefore, the present study summarized the current research on CMD by applying a bibliometric approach, with a focus on: (i) the most influential countries, institutions, authors, and journals, as well as their impact and contribution to this field, and (ii) the research status, hotspots, and development trends in this field via an analysis of keywords and co-cited references. The purpose of this study is to develop an overall structure of the field of CMD and to identify research hotspots and trends to assist and guide future directions.

## Materials and methods

### Data sources

**Fig. 1:**
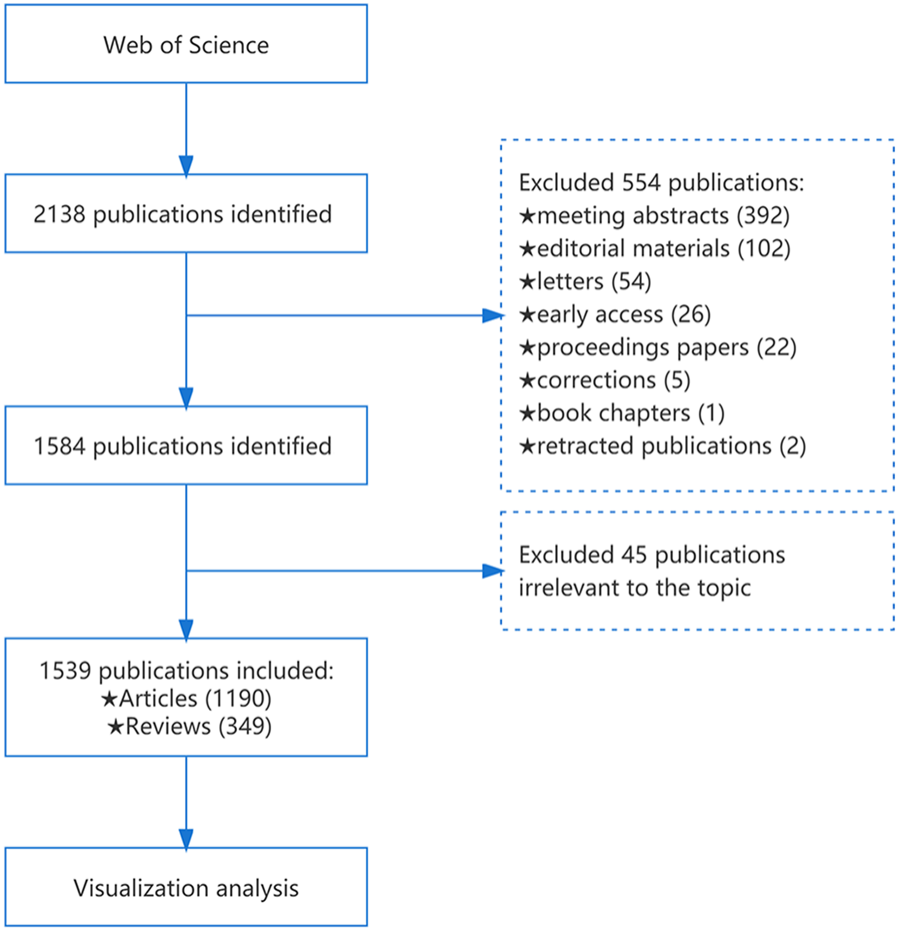
Flow diagram of literature selection

A literature search regarding CMD was conducted on the Web of Science Core Collection (WoSCC). The search strategy was as follows: TS = (“coronary microvascular” OR “coronary microcirculation” OR “coronary artery microcirculation” OR “coronary artery microvascular”) AND TS = (disorder* OR disturbance* OR disease* OR dysfunction*); literature type = article or review. The time span was between 2002 and 2022, and there was no language restriction. All data were downloaded in TXT format. To avoid changes due to daily updates to the database, all publication searches and data extractions were conducted on July 3, 2022. The flow chart of the study is presented in Fig. 1.

### Data analysis

Visualization network maps of countries, institutions, authors, and co-cited authors were built using VOSviewer (version 1.6.18) to analyze general information in the field of CMD. CiteSpace (version 5.8.R3) was used not only for keyword co-occurrence analysis, keyword cluster analysis, and keyword burst but also for the construction of a dual-map overlay of journals and a timeline view of co-cited references, allowing us to understand the status and predict the frontiers and hotspots of research in this field.

## Results

### Growing trends of publications

**Fig. 2:**
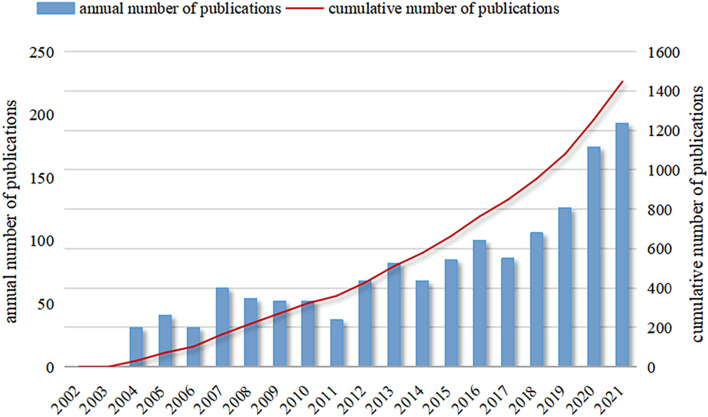
Annual and cumulative number of publications related to coronary microvascular dysfunction

A total of 1539 publications (1190 articles and 349 reviews) met the inclusion criteria. The annual distribution of publications on CMD is shown in Fig. 2. The first article on CMD was published in 2004, with no papers being published in 2002 and 2003. The annual number of publications slightly declined at some timepoints between 2004 and 2021; however, an overall upward trend was observed (from 0 in 2002 to 193 in 2021). As of July 3, the number of publications in 2022 had reached 91. In general, CMD-related research has entered a period of rapid development and has recently received increasing attention from researchers.

### Analysis of countries and institutions

**Fig. 3:**
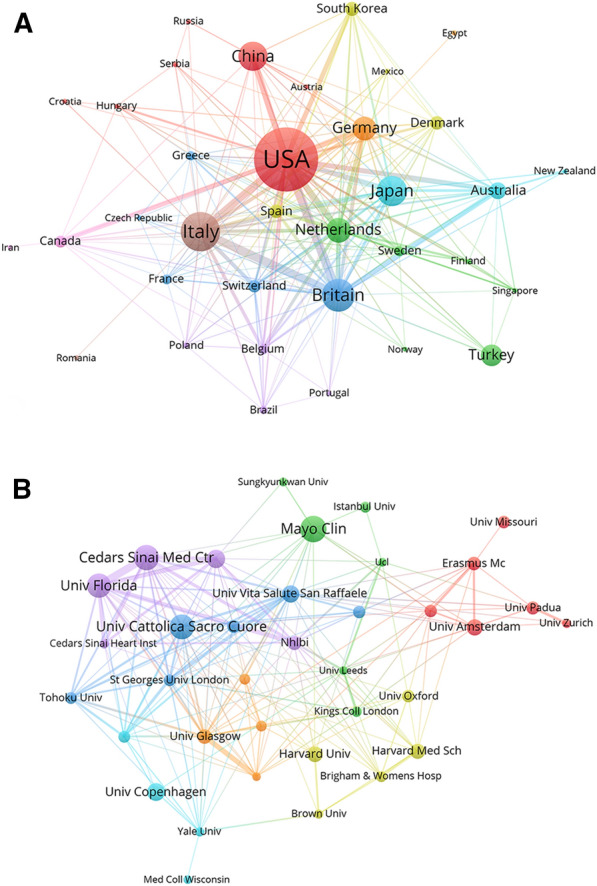
The visualization network map of collaboration between countries and institutions. **A** Collaboration network map of countries. **B** Collaboration network map of institutions

The 1539 publications on CMD were from 57 different countries and 1643 different institutions. The visualization network maps show the number of articles published by different countries and institutions and the cooperation between them. The size of the nodes represents the number of published literature; the greater the size, the larger the number of publications. The links represent the association between countries or institutions, with a thicker link implying more cooperation. Overall, 35 countries had more than five articles, and 37 institutions had more than 15 articles (Fig. 3A and B). Publications on CMD were unevenly distributed across different countries. As presented in Table 1, the United States of America (USA) showed the highest output with 515 papers, accounting for 33.5% of the total, followed by Italy (239 papers), the United Kingdom (UK) (183 papers), Japan (158 papers), and China (151 papers). The UK had the highest average number of citations, indicating a strong academic reputation in the field of CMD. According to Table 2, three out of the top five institutions with the highest number of published papers were from the USA. The Mayo Clinic from the USA had the most publications. Furthermore, the University of Florida from the USA had the most citations on average, ranking fourth with respect to the number of published papers.

**Table 1:** Top 5 productive countries related to coronary microvascular dysfunction

Rank	Country	Publications	Citations	Average Citations
1	USA	515	19,101	37
2	Italy	239	8610	36
3	UK	183	8006	44
4	Japan	158	3081	20
5	China	151	1114	7

USA, the United States of America; UK, the United Kingdom

**Table 2:** Top 5 productive institutions related to coronary microvascular dysfunction

Rank	Institution	Publications	Citations	Average Citations	Country
1	Mayo Clinic	50	317	6	USA
2	Cedars Sinai Medical Center	48	940	20	USA
3	Universita Cattolica del Sacro Cuore	47	810	17	Italy
4	University of Florida	46	2002	44	USA
5	Baskent University	42	859	20	Turkey

USA, the United States of America

Extensive collaboration between countries and institutions promotes the development of a discipline. In the field of CMD, the cooperation between countries was relatively close, particularly the USA, which collaborated the most with other countries. In addition, a number of institutions exhibited active and close cooperation, especially institutions in the USA such as the University of Florida.

### Analysis of authors and co-cited authors

**Fig. 4:**
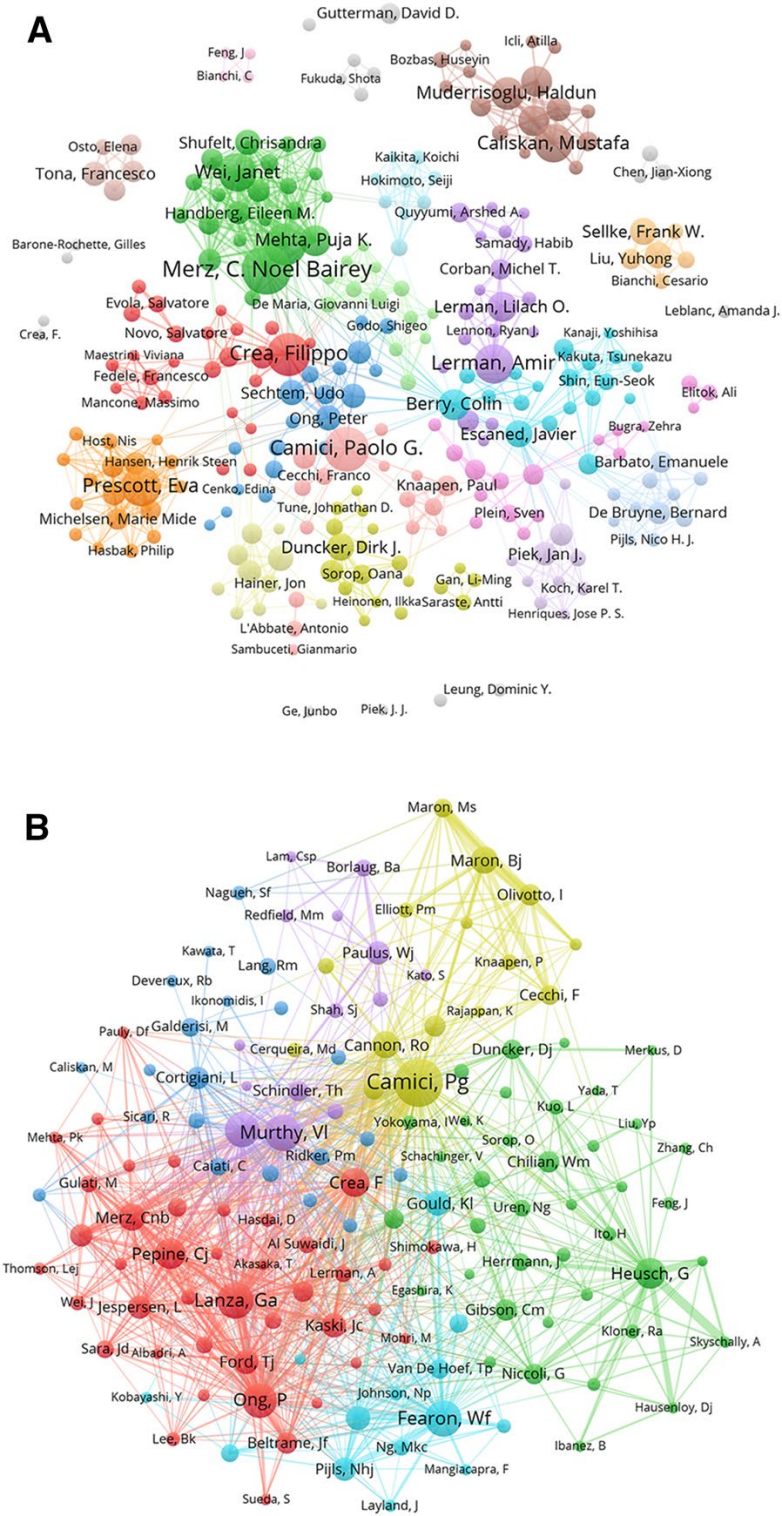
The visualization network map of cooperation and co-citation of authors. **A** Cooperation network map of authors. **B** Co-citation network map of authors

A total of 7629 authors contributed to these 1539 articles, with 29,731 co-cited authors. An analysis of authors contributes to understanding the representative researchers and core research trends in a field. Additional file 1 lists the top eight most productive authors with more than 30 publications as well as the most frequently co-cited authors with more than 250 citations. Merz C. N. B., an American researcher who focused on investigating CMD in women, was the most productive author, with 48 papers [19–22]. The second most productive author was Camici P. G. from Italy, with 41 papers, who was concerned with research on CMD in hypertrophic cardiomyopathy [23–25]. The third most productive author was another Italian researcher, Crea F., who published 38 articles and focused on the relationship between CMD and either myocardial infarction or percutaneous coronary interventions [26–29]. Figure 4A shows the collaborative relationships among authors with more than five publications, indicating that authors formed relatively stable collaborative groups.

The top eight most frequently co-cited authors were all from Western countries, with four from the USA, two from Germany, and two from Italy. Among the top eight most frequently co-cited authors, Camici P. G. ranked second with respect to the number of publications and had the most co-citations (535 times), followed by Fearon W. F. (342 co-citations) and Murthy V. L. (341 co-citations). Figure 4B shows the co-citation relationships between co-cited authors with more than 50 co-citations.

### Analysis of journals and co-cited journals

Overall, CMD-related articles were published in 382 journals, with the top 10 most prolific journals being presented in Table 3. According to 2021 journal citation reports, 2/5 of the top 10 most prolific journals were classified to be in Quartile 1 (Q1). The *International Journal of Cardiology* published the most papers (*n* = 76), followed by the *American Journal of Physiology-Heart and Circulatory Physiology* (*n* = 43) and the *European Heart Journal* (*n* = 32). Among the top 10 most prolific journals, *Circulation* had the highest impact factor (IF) (IF = 39.918), followed by the *European Heart Journal* (IF = 35.855).

**Table 3:** Top 10 productive journals related to coronary microvascular dysfunction

Rank	Journal	Publications	JCR	IF (2021)
1	International Journal of Cardiology	76	Q2	4.039
2	American Journal of Physiology-Heart and Circulatory Physiology	43	Q2	5.125
3	European Heart Journal	32	Q1	35.855
4	Journal of the American College of Cardiology	30	Q1	27.203
5	Atherosclerosis	29	Q1	6.847
6	Circulation	28	Q1	39.918
7	Microcirculation	28	Q4	2.679
8	Cardiovascular Research	27	Q1	13.081
9	Journal of Nuclear Cardiology	27	Q2	3.872
10	Journal of the American Heart Association	26	Q2	6.106

JCR, journal citation reports; IF, impact factor

In total, 4191 co-cited journals were identified and Table 4 summarizes the top 10 journals with the highest co-citations. *Circulation* had the most co-citations (10,084 times), followed by the *Journal of the American College of Cardiology* (7177 times) and the *European Heart Journal* (3658 times). Among co-cited journals, the *New England Journal of Medicine* had the highest IF (IF = 176.079). In addition, 7/10 of the most co-cited journals were in Q1, and five of them were listed as among the top 10 most prolific journals.

**Table 4:** Top 10 co-cited journals related to coronary microvascular dysfunction

Rank	Journal	Citations	JCR	IF (2021)
1	Circulation	10,084	Q1	39.918
2	Journal of the American College of Cardiology	7177	Q1	27.203
3	European Heart Journal	3658	Q1	35.855
4	New England Journal of Medicine	2546	Q1	176.079
5	American Journal of Cardiology	2363	Q3	3.133
6	Circulation Research	2007	Q1	23.213
7	American Journal of Physiology-Heart and Circulatory Physiology	1938	Q1	5.125
8	International Journal of Cardiology	1414	Q2	4.039
9	American Heart Journal	1220	Q2	5.099
10	Cardiovascular Research	1115	Q1	13.081

JCR, journal citation reports; IF, impact factor

**Fig. 5:**
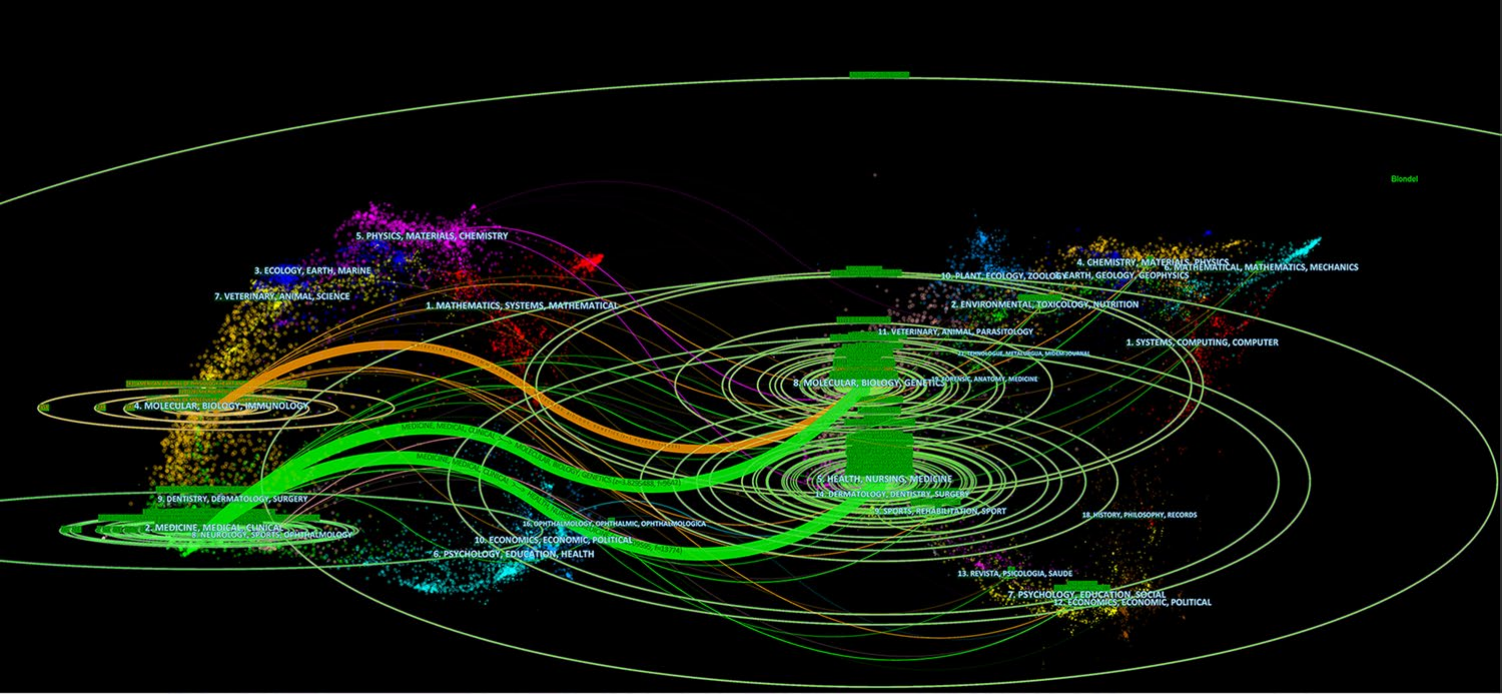
The dual-map overlay of journals on coronary microvascular dysfunction

Furthermore, a dual-map overlay of journals was constructed to show the citation relationship of the journals (Fig. 5). The citing journals are presented on the left, whereas the cited journals are on the right, with the colored line between them indicating the citation paths. The orange-colored path indicates that the articles belonging to Molecular/Biology/Genetics journals were primarily cited by those in Molecular/Biology/Immunology journals. On the other hand, the green-colored citation paths indicate that the articles from Molecular/Biology/Genetics and Health/Nursing/Medicine journals were primarily cited by those published in Medicine/Medical/Clinical journals.

### Analysis of keywords

**Fig. 6:**
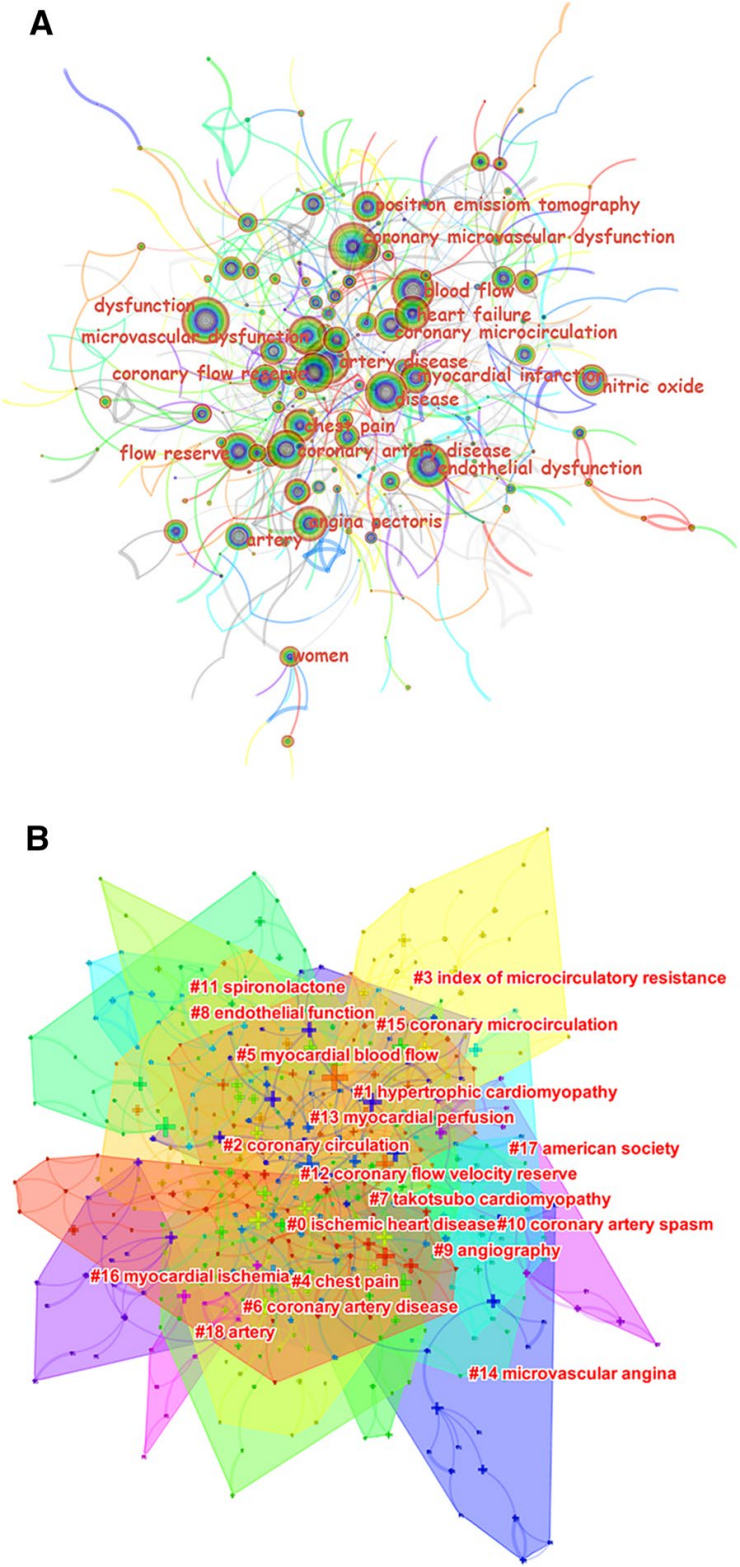
The visualization network map of keywords. **A** Network map of keyword co-occurrence. **B** Network map of keyword clusters

Keywords with similar meanings were combined prior to analysis. Figure 6A presents the network map of keyword co-occurrence. The larger the node is, the more frequently a keyword occurs; furthermore, the thicker the line between the nodes is, the more frequently the two keywords appear together. The keywords with the highest frequency of occurrence are presented in Table 5. After excluding the keywords consistent with the theme of our study, the most frequent keywords were as follows, covering the pathophysiology, clinical symptoms, diagnostic methods, and clinical implications of CMD: “coronary flow reserve” (183 times), “flow reserve” (180 times), “heart failure” (180 times), “endothelial dysfunction” (173 times), “angina pectoris” (160 times), “chest pain” (155 times), “nitric oxide” (129 times), “positron emission tomography” (118 times), and “myocardial infarction” (117 times).

**Table 5:** Top 20 high-frequency keyword related to coronary microvascular dysfunction

Rank	Keyword	Count	Rank	Keyword	Count
1	Coronary microvascular dysfunction	364	11	Coronary artery disease	162
2	Artery disease	239	12	Angina pectoris	160
3	Dysfunction	215	13	Chest pain	155
4	Microvascular dysfunction	189	14	Nitric oxide	129
5	Coronary flow reserve	183	15	Coronary microcirculation	125
6	Flow reserve	180	16	Positron emission tomography	118
7	Heart failure	180	17	Myocardial infarction	117
8	Blood flow	177	18	Risk	110
9	Endothelial dysfunction	173	19	Cardiac syndrome x	103
10	Disease	163	20	Risk factor	101

Keyword cluster analysis was conducted to reveal the current hotspots and basic knowledge structure of CMD-related research. The modularity Q of the cluster was 0.6963, indicating a significant cluster structure. The silhouette S was 0.856, suggesting convincing network homogeneity. Overall, 19 clusters were obtained (Fig. 6B). Clusters #2, #6, #15, and #18 were consistent with the theme of our study. Cluster #17 was related to research institutions and participants. The remaining clusters were categorized into the following five groups: (i) cluster #8, which focused on the pathophysiology of CMD, including “endothelial function”; (ii) cluster #4, which focused on the clinical symptoms of CMD, such as “chest pain”; (iii) clusters #3, #5, #12, #13, and #9, which focused on the diagnostic methods for CMD, including “index of microcirculatory resistance,” “coronary flow velocity reserve,” and “angiography”; (iv) clusters #0, #1, #7, #10, #14, and #16, which focused on CMD-related clinical implications, including “hypertrophic cardiomyopathy,” “takotsubo cardiomyopathy,” “microvascular angina,” and “heart failure”; and (v) cluster #11, which focused on the management of CMD, including “spironolactone.”

**Fig. 7:**
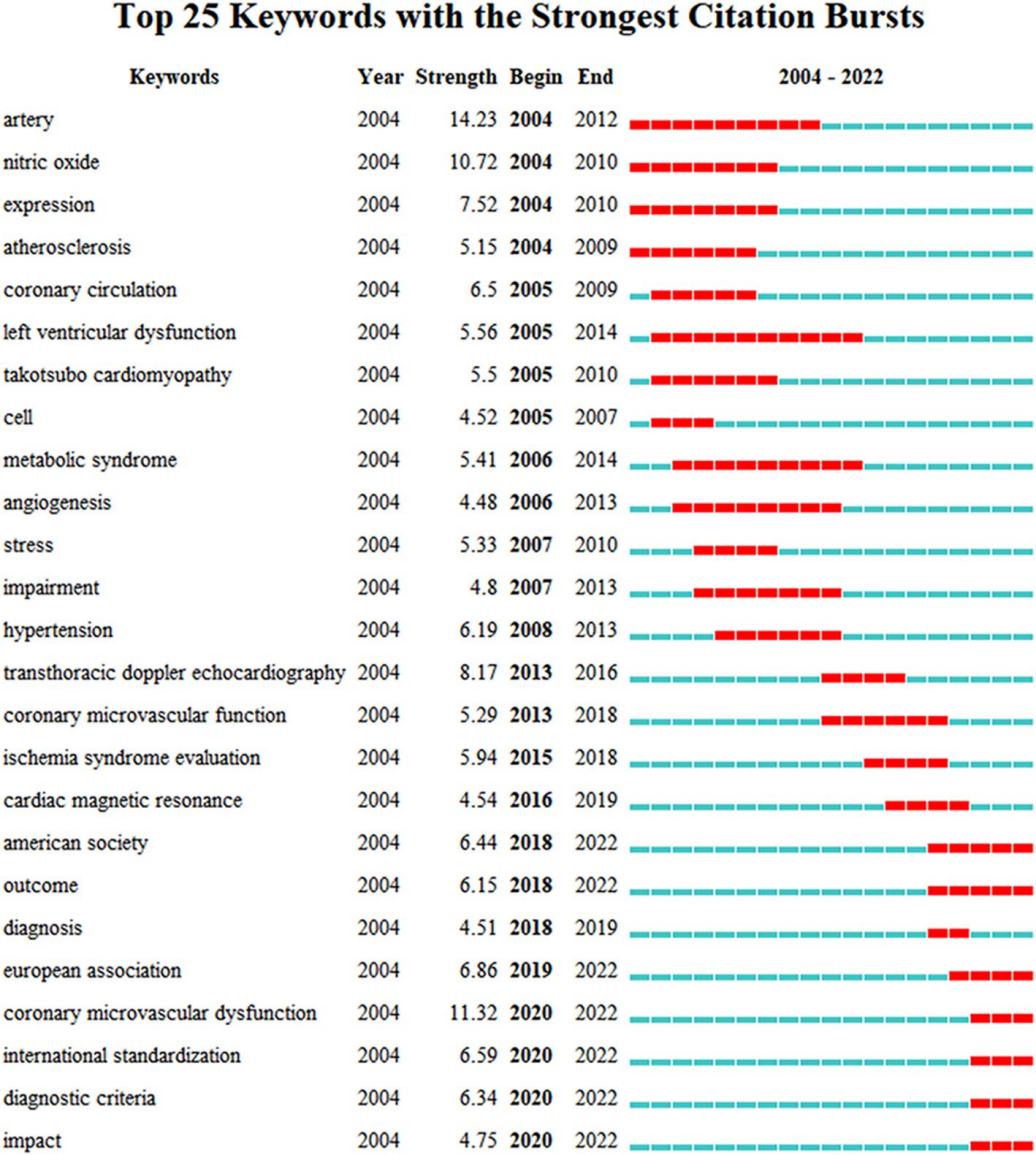
Top 25 burst keywords related to coronary microvascular dysfunction

The keyword burst more comprehensively reflected the trends and frontiers of research on CMD and helped in predicting future research directions. Figure 7 depicts the top 25 burst keywords, with their start and end years as well as burst strengths. The blue line represents the timeline from 2004 to 2022, whereas the red line indicates the keyword burst during this period. It could be observed that the keywords were related to pathophysiology (nitric oxide), diagnostic methods (transthoracic Doppler echocardiography and cardiac magnetic resonance), and clinical implications (left ventricular dysfunction, takotsubo cardiomyopathy, metabolic syndrome, and hypertension). Notably, the keyword “outcome” had persisted from 2018 to 2022, implying that studies concerning the prognosis of CMD have continued to receive attention over recent years. The terms “international standardization” and “diagnostic criteria” indicated that the development of international guidelines for the diagnosis and treatment of CMD might be a future research direction.

### Analysis of co-cited references

A total of 46,826 references were obtained. An analysis of the most co-cited references allowed researchers to appreciate research hotspots in a field. Additional file 2 shows the top 10 most co-cited references. The paper by Camici et al. titled “Coronary Microvascular Dysfunction,” which was published in the *New England Journal of Medicine* in 2007, was the most frequently cited article (309 citations). This paper reviewed examination methods, clinical classifications, and pathogenetic mechanisms of CMD [30]. The second most co-cited reference (178 times) was the article by Pepine et al. titled “Coronary Microvascular Reactivity to Adenosine Predicts Adverse Outcome in Women Evaluated for Suspected Ischemia: Results from the NHLBI Women's Ischemia Syndrome Evaluation (WISE).” This article reported that in women without obstructive coronary artery diseases, a lower coronary flow reserve led to a higher risk of major adverse events, including mortality, nonfatal stroke, nonfatal myocardial infarction, and hospitalization for heart failure, suggesting that dysfunctional coronary microvessels might be novel targets for diagnostic and therapeutic strategies in patients with ischemic heart disease [31]. The article by Crea et al. titled “Coronary Microvascular Dysfunction: an Update,” which reviewed risk factors for CMD and recent advances in treatment strategies for CMD, was the third most co-cited reference (139 citations) [32]. Among the top 10 most frequently co-cited references, the article published by Ong et al. titled “International Standardization of Diagnostic Criteria for Microvascular Angina” had the highest number of average citations per year; this article provided a standardized guideline for the mechanism, diagnosis, prognosis, and clinical trial studies of microvascular angina [33].

**Fig. 8:**
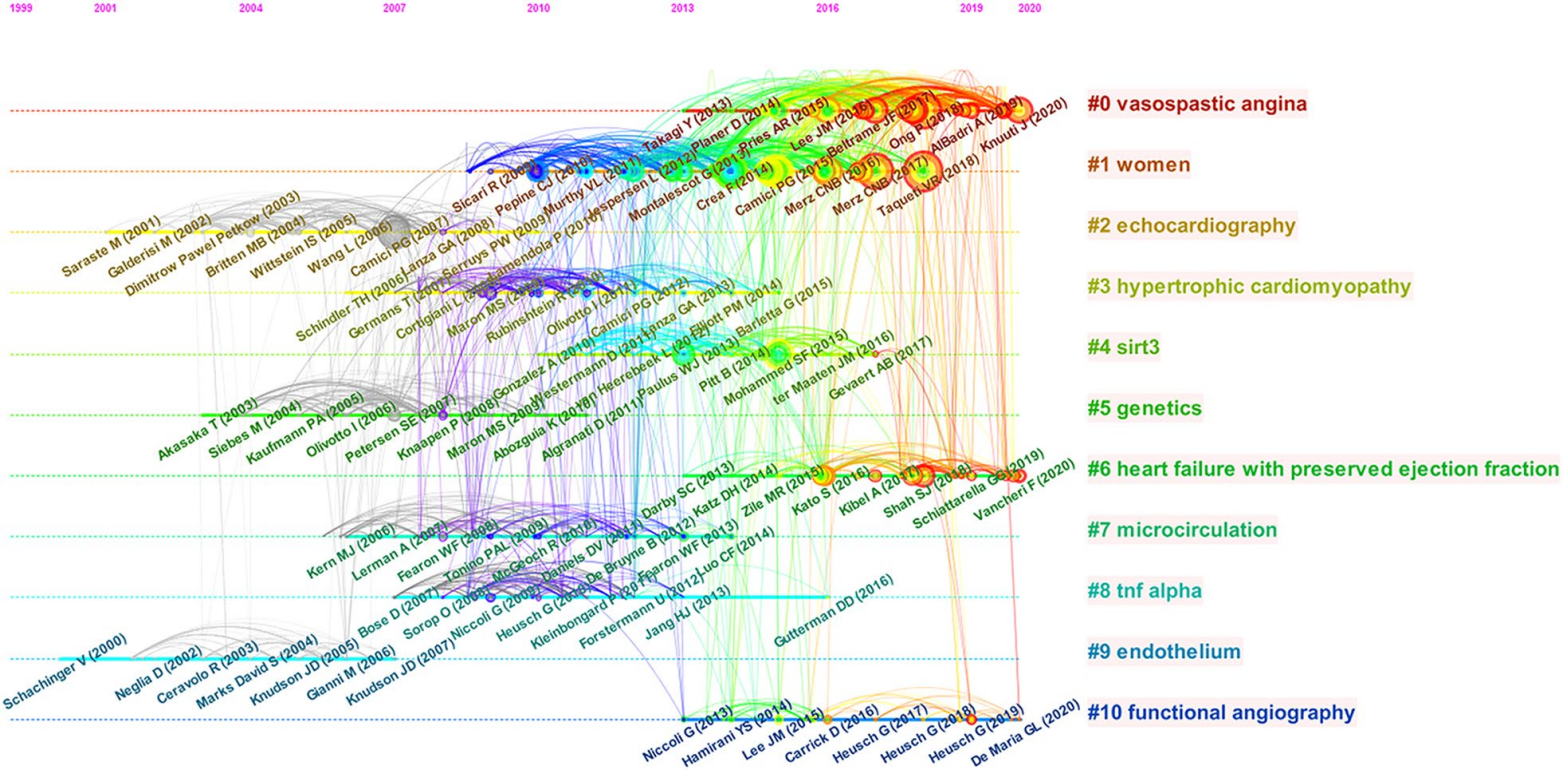
Timeline view of co-cited references

Figure 8 presents a timeline view of co-cited references, showing the distribution of themes and their changes with time. Early research in this field primarily focused on “endothelium,” “echocardiography,” and “genetics.” However, these themes were gradually replaced by new themes over time. Recently, researchers had focused on “vasospastic angina,” “women,” “heart failure with preserved ejection fraction,” and “functional angiography,” particularly the relationship between CMD and vasospastic angina as well as between CMD and female sex.

## Discussion

### General information

The research progress in the field of CMD could be estimated based on the number and trend of annual publications [34]. In general, the number of publications in this field had gradually increased over the past two decades. Relatively few articles were published from 2004 to 2019, indicating that research on CMD remains in its infancy. Since 2019, publications on CMD have entered a period of rapid growth, suggesting that research on CMD has received increasing attention over recent years.

Among numerous countries, the USA has made the most substantial contribution to this field. Three out of the top five most productive institutions (Mayo Clinic, Cedars Sinai Medical Center, and University of Florida) and four out of the top 10 most productive researchers are from the USA, making it the most productive country, with a total of 515 articles. In addition, the collaboration between the USA and other countries as well as between highly productive American institutions was mostly close, which had contributed greatly to the promotion of international communication and academic development in this field. As for the other countries and institutions, stronger collaboration and academic communication were required to facilitate development and progress in this field.

An analysis of author collaborative networks aids in understanding influential researchers and the collaborative relationship between researchers [35]. The most influential author was identified to be Camici P. G. from Italy, who ranked first in terms of co-citations and second with respect to the number of publications. Most articles by Camici P. G. were published in the top journals in the cardiovascular field, including the *European Heart Journal*, *Circulation*, and *Journal of the American College of Cardiology*. Among the publications of Camici P. G., the article titled “Coronary Microvascular Dysfunction,” which was published in the *New England Journal of Medicine* in 2007, was the most influential. This article summarized the four major types of CMD according to the clinical setting in which CMD occurs and the pathogenesis of CMD, including iatrogenic CMD, CMD in obstructive coronary artery disease, CMD in myocardial disease, and CMD without myocardial and obstructive coronary artery diseases [30]. In 2014, Camici P. G. and his partner, Crea F., updated this review in an article titled “Coronary Microvascular Dysfunction: an Update,” published in the *European Heart Journal*. This article reviewed the pathogenesis and treatment of CMD and suggested that the underlying etiologies of CMD should be considered in clinical management, whether obstructive coronary artery disease or myocardial disease were present [32].

In addition, the *European Heart Journal*, *Circulation*, and *Journal of the American College of Cardiology* were all in Q1 and were present in all of the following three lists: the top 10 most prolific journals, the top 10 most co-cited journals, and the source journals for the top 10 most co-cited references. Thus, the *European Heart Journal*, *Circulation*, and *Journal of the American College of Cardiology* are the most mainstream and authoritative journals in the field of CMD.

### Hotspots and frontiers

Based on the analysis of keywords and references, the hotspots and frontiers of research are summarized as follows:

#### Pathophysiology of CMD

Related keywords mainly included “endothelial dysfunction” and “nitric oxide.” Endothelial dysfunction is considered to be an important pathogenic factor for CMD. Normally, vascular endothelial cells can release vasodilator substances, such as nitric oxide, to modulate blood flow. The decreased production or reduced bioavailability of nitric oxide caused by impaired endothelial cells results in impaired vasodilation function. The impaired endothelial cells may also release vasoconstrictor substances that can reduce blood flow, such as endothelin, thromboxane A2, prostaglandin H2, and superoxide. Furthermore, the reduced bioavailability of nitric oxide leads to microvascular rarefaction and remodeling by causing angiogenic incompetence and increasing collagen deposition, which prompts functional and structural abnormalities of microcirculation, thus increasing the risk of myocardial ischemia [36, 37]. Rahman et al. proposed the existence of two distinct CMD endotypes based on physiological assessment in a catheter laboratory—namely, structural CMD and functional CMD [38]. Functional CMD is characterized by an attenuated vasodilatory reserve, which may be due to endothelial dysfunction, smooth muscle cell dysfunction, and sympathetic dysfunction [37, 39]. These may be attributed to an impairment in sirtuin 3-mediated endothelial cell metabolism, dysregulation of the endothelial-1/endothelin A receptor system, and abnormalities of the synthetic phenotype of viable smooth muscle cells responsible for radial contraction [40–42]. Structural abnormalities in CMD are mainly manifested by luminal narrowing of the microvessels, perivascular fibrosis, and capillary rarefaction [8], which are also considered to play a fundamental pathogenic role in hypertrophic cardiomyopathy and heart failure [24]. In addition, hypertension, obesity, and diabetes have been identified as risk factors for CMD [43–45]. A previous study on pigs confirmed that diabetes could lead to CMD by causing cardiac microvascular rarefaction [46]. Research on pathophysiology allows for the possibility of preventing and treating CMD and is, hence, at the forefront.

#### Symptoms and diagnostic methods of CMD

The major keywords primarily included “chest pain,” “coronary flow reserve,” “index of microcirculatory resistance,” “angiography,” “coronary flow velocity reserve,” “myocardial perfusion,” “positron emission tomography,” “transthoracic Doppler echocardiography,” and “cardiac magnetic resonance.” Chest pain is the primary clinical manifestation of CMD [47], and approximately 30–60% of patients with CMD have been reported to have angina pectoris [48]. Currently, the diagnosis of CMD involves invasive and non-invasive techniques. Invasive methods, including the use of intracoronary Doppler-tipped guidewire, thermodilution techniques, and coronary reactivity testing, are the gold standard for CMD detection, which can measure the index of microvascular resistance and coronary flow reserve [2]. Non-invasive techniques can evaluate non-endothelial-dependent coronary microvascular function if there is no epicardial vessel obstruction [49]. Positron emission tomography represents a non-invasive imaging method that has recently been widely used to quantify myocardial blood flow, and its accuracy has been demonstrated by both animal and human experiments [50, 51]. Cardiovascular magnetic resonance imaging, another non-invasive detection technique for diagnosing CMD, has attracted increasing attention. Kellman et al. optimized the dual sequence for myocardial perfusion cardiovascular magnetic resonance imaging and arterial input function measurement, which was used for the quantitative detection of myocardial blood flow [52]. Transthoracic Doppler echocardiography has been applied to the assessment of coronary flow velocity ratios and has been shown to predict adverse outcomes in patients with angina and no obstructive coronary artery disease [50]. Therefore, the diagnosis of CMD remains a major research topic in this field because of the lack of an accurate, direct, and economical detection method for assessing microvascular function.

#### Clinical implications of CMD

Keywords in this category mainly included “heart failure,” “cardiac syndrome X,” “myocardial infarction,” “hypertrophic cardiomyopathy,” “takotsubo cardiomyopathy,” and “microvascular angina.” CMD is strongly associated with various coronary artery diseases [8]. Previous studies reported that approximately 40–70% of patients who underwent diagnostic coronary angiography for chest pain had no obstructive coronary artery disease, which was considered to be related to microvascular dysfunction [11, 12, 53]. Furthermore, the prevalence of CMD in patients with HFpEF had been reported to be as high as 75%, with CMD having been shown to be positively associated with NTproBNP, cardiac dysfunction, and systemic endothelial dysfunction; therefore, it might be a potential risk predictor and a novel therapeutic target for HFpEF [13]. Further studies revealed that subendocardial ischemia might be one of the pathophysiological mechanisms underlying CMD-related HFpEF [54, 55]. Reduced capillary density and vascular remodeling and fibrosis lead to a decrease in the coronary blood flow reserve, which in turn results in the development of hypertrophic cardiomyopathy and is closely associated with adverse prognosis [56–59]. Takotsubo cardiomyopathy, also referred to as stress-induced cardiomyopathy or broken heart syndrome, presents as acute, transient, and reversible systolic dysfunction of the left ventricle [60, 61], and is strongly associated with CMD. According to some studies, patients with takotsubo cardiomyopathy frequently have impaired myocardial perfusion and reduced coronary flow reserve [62, 63]. Furthermore, CMD can lead to the “no-reflow phenomenon” in patients with acute myocardial infarction, and the mechanisms involve damage to the arterial walls or obstruction in the microcirculation [64]. In addition, CMD in other diseases such as autoimmune rheumatic diseases and liver diseases has gained increasing attention [65–68]. The association between these clinical diseases and CMD has been shown in multiple studies; nonetheless, the deeper mechanisms still require extensive research.

#### Prognosis and management of CMD

The related keywords included “outcome” and “spironolactone.” Compared with people with normal perfusion, a reduced coronary flow reserve in patients means a four-to-fivefold increase in death and a three-to-fivefold increase in MACEs [14, 69, 70]; the lower the coronary flow reserve, the higher the risk of adverse outcomes [71]. Previous studies showed the substantial impact of CMD on the prognosis of patients with myocardial infarction [72–74]. In a cohort study involving 309 patients with ST-segment elevation myocardial infarction (STEMI) who were successfully revascularized, an index of microvascular resistance above 40 U signified a substantially increased risk of cardiac mortality or readmission for heart failure and was an independent predictor [75]. However, another study suggested that the bimodal shape of the thermodilution curve, rather than the index of microvascular resistance value itself, indicated an increased risk of MACEs within six months, and this might be a result of capillary obstructions [76]. A systematic review of 1094 patients further suggested that the index of hyperemic microvascular resistance and the index of microcirculatory resistance in patients with STEMI after PCI aided in the early identification and early risk stratification of severe CMD and were prognostic indicators of MACEs [77]. In addition, CMD could be a prognostic indicator of MACEs in patients with hypertrophic cardiomyopathy, obesity, aortic stenosis, or chronic kidney disease [59, 78–80]. Therefore, CMD may serve as a crucial therapeutic target. Current studies have found that the coronary flow reserve can be enhanced by drugs targeting the renin–angiotensin–aldosterone system, statins, calcium channel blockers, and ranolazine to minimize MACEs [81–86]. Some traditional Chinese medicines have also been used to treat CMD, including Shenmai injection and Shexiang Tongxin dropping pills, which may improve coronary microvascular function, endothelial function, and microvascular structure abnormalities by reducing oxidative stress, inflammatory response, and apoptosis [87, 88]. In addition, CD34+ stem/progenitor cell therapy and neuropeptide-Y_1_ receptor antagonists have been discovered to possess therapeutic potential for CMD [89, 90]. Targeting the regulation of coronary microvascular dysfunction to improve clinical prognosis will be one of the difficulties and hotspots in the field of CMD in the future.

The results of the keyword burst notably indicated that “international standardization” and “diagnostic criteria” had recently been the major topics. Currently, the identification, diagnosis, and treatment of CMD are scattered in the guidelines for other diseases, and no standardized guidelines for the diagnosis and management of CMD have been developed. Therefore, several relevant studies and summaries are required.

## Strengths and limitations

To date, the present study is the first bibliometric analysis in the field of CMD in which the most influential authors, countries, institutions, and journals as well as the research hotspots and development trends in this field were identified. However, this study has some limitations. First, publications were obtained from the WoSCC only, and other databases were absent. Nevertheless, the WoSCC is recognized as the most authoritative, comprehensive, and multidisciplinary database and is the most commonly used database for bibliometric analysis. Therefore, the articles included in this study were relatively comprehensive. Second, some recent high-quality publications were less cited, and their influence might have been underestimated. Thus, a future update of the bibliometric analysis in the field of CMD is necessary.

## Conclusions

Overall, the number of annual publications in the field of CMD showed an upward trend from 2002 to 2022. The USA was the most influential country and had established stable partnerships with several other countries. Camici P. G. was the most influential author. With respect to the number of publications and co-citations, the *European Heart Journal*, *Circulation*, and *Journal of the American College of Cardiology* were the most authoritative journals in this field. The research hotspots were primarily related to the pathophysiology, diagnostic methods, clinical implications, and management of CMD. Specifically, regarding the pathophysiology of CMD, endothelial dysfunction as well as reduced nitric oxide production or bioavailability played critical roles in CMD development. As for diagnosis, invasive techniques were gradually replaced by non-invasive techniques, and positron emission tomography was currently the most widely used imaging method. In terms of CMD-related clinical implications, microvascular angina, hypertrophic cardiomyopathy, and heart failure had been identified as the hotspots over recent years. Furthermore, CMD had been shown to be associated with an increased risk of adverse outcomes; thus, treatment and management to improve clinical prognosis are at the forefront. However, international standards for the diagnosis and management of CMD are still currently lacking, which may be a future research direction.

## Supplementary Information

**Additional file 1. **Top 8 productive authors and co-cited authors related to coronary microvascular dysfunction.

**Additional file 2. **Top 10 co-cited references related to coronary microvascular dysfunction.

## Data Availability

All data generated or analyzed during this study are included in this published article and its additional information files.
