# Supplementary material for: Global trends and frontiers in research on coronary microvascular dysfunction: a bibliometric analysis from 2002 to 2022
Source: Eur J Med Res. 2022 Nov 5;27:233. doi: 10.1186/s40001-022-00869-8 (PMC9636644; doi:10.1186/s40001-022-00869-8)
Supplement: Supplementary file 1 — Additional file 1. Top 8 productive authors and co-cited authors related to coronary microvascular dysfunction. [file 40001_2022_869_MOESM1_ESM.docx]

| **Rank** | **Author** | **Publications** | **Country** | **Rank** | **Co-cited Author** | **Citations** | **Country** |
| --- | --- | --- | --- | --- | --- | --- | --- |
| 1 | Merz C. N. B. | 48 | USA | 1 | Camici P. G. | 535 | Italy |
| 2 | Camici P. G. | 41 | Italy | 2 | Fearon W. F. | 342 | USA |
| 3 | Crea F. | 38 | Italy | 3 | Murthy V. L. | 341 | USA |
| 4 | Pepine C. J. | 37 | USA | 4 | Lanza G. A. | 338 | Italy |
| 5 | Lerman A. | 33 | USA | 5 | Taqueti V. R. | 314 | USA |
| 6 | Prescott E. | 33 | Denmark | 6 | Ong P. | 308 | Germany |
| 7 | Caliskan M. | 32 | Turkey | 7 | Pepine C. J. | 257 | USA |
| 8 | Wei J. | 32 | USA | 8 | Heusch G. | 254 | Germany |

**Additional file 1** Top 8 productive authors and co-cited authors related to coronary microvascular dysfunction.

USA, the United States of America.
