# Supplementary material for: Global trends and frontiers in research on coronary microvascular dysfunction: a bibliometric analysis from 2002 to 2022
Source: Eur J Med Res. 2022 Nov 5;27:233. doi: 10.1186/s40001-022-00869-8 (PMC9636644; doi:10.1186/s40001-022-00869-8)
Supplement: Supplementary file 2 — Additional file 2. Top 10 co-cited references related to coronary microvascular dysfunction. [file 40001_2022_869_MOESM2_ESM.docx]

**Additional file 2** Top 10 co-cited references related to coronary microvascular dysfunction.

| **Rank** | **Title** | **Corresponding Authors** | **Journal** | **IF** | **Publication Year** | **Total Citations** | **Average Citations** |
| --- | --- | --- | --- | --- | --- | --- | --- |
| 1 | Coronary Microvascular Dysfunction | Camici P. G. | New England Journal of Medicine | 176.079 | 2007 | 309 | 19 |
| 2 | Coronary Microvascular Reactivity to Adenosine Predicts Adverse Outcome in Women Evaluated for Suspected Ischemia Results From the National Heart, Lung and Blood Institute WISE (Women's Ischemia Syndrome Evaluation) Study | Pepine C. J. | Journal of the American College of Cardiology | 27.203 | 2010 | 178 | 14 |
| 3 | Coronary Microvascular Dysfunction: an Update | Crea F. | European Heart Journal | 35.855 | 2014 | 139 | 15 |
| 4 | Effects of Sex on Coronary Microvascular Dysfunction and Cardiac Outcomes | Di Carli M. F. | Circulation | 39.918 | 2014 | 128 | 14 |
| 5 | A Novel Paradigm for Heart Failure With Preserved Ejection Fraction Comorbidities Drive Myocardial Dysfunction and Remodeling Through Coronary Microvascular Endothelial Inflammation | Paulus W. J. | Journal of the American College of Cardiology | 27.203 | 2013 | 125 | 13 |
| 6 | Stable Angina Pectoris With No Obstructive Coronary Artery Disease is Associated With Increased Risks of Major Adverse Cardiovascular Events | Jespersen L. | European Heart Journal | 35.855 | 2012 | 123 | 11 |
| 7 | Novel Index for Invasively Assessing the Coronary Microcirculation | Fearon W. F. | Circulation | 39.918 | 2003 | 120 | 6 |
| 8 | Coronary Microvascular Dysfunction and Prognosis in Hypertrophic Cardiomyopathy | Cecchi F. | New England Journal of Medicine | 176.079 | 2003 | 117 | 6 |
| 9 | International Standardization of Diagnostic Criteria for Microvascular Angina | Ong P. | International Journal of Cardiology | 4.039 | 2018 | 107 | 21 |
| 10 | Coronary Microvascular Dysfunction: Mechanisms and Functional Assessment | Rimoldi O. | Nature Reviews Cardiology | 49.421 | 2015 | 98 | 12 |

IF, Impact Factor.
